# BMI Impact on Readmissions for Patients Undergoing Robot-Assisted Radical Prostatectomy: A Monocentric, Single-Surgeon Serial Analysis of 500 Cases

**DOI:** 10.3390/jcm12123908

**Published:** 2023-06-07

**Authors:** Mahmoud Farzat, Ismail Sharabaty, Christian Tanislav, Yaman Alsaid, Florian M. Wagenlehner

**Affiliations:** 1 Department of Robotic Urology, Diakonie Klinikum Siegen, Academic Teaching Hospital, University of Bonn, 53127 Bonn, Germany; ismail.sharabaty@diakonie-sw.de; 2 Department of Urology, Pediatric Urology and Andrology, Justus-Liebig University of Giessen, 35390 Giessen, Germany; florian.wagenlehner@chiru.med.uni-giessen.de; 3 Department of Geriatric and Neurology, Diakonie Klinkum Siegen, Academic Teaching Hospital, University of Bonn, 53127 Bonn, Germany; christian.tanislav@diakonie-sw.de; 4 Department of Urology, St. Josef Hospital Engelskirchen, 51766 Engelskirchen, Germany; yamanalsaid@gmail.com

**Keywords:** prostate cancer, RARP, BMI, obesity, readmissions

## Abstract

Due to more difficult intraoperative courses, elevated rates of case abortion and unfavored postoperative outcomes in obese patients, urologists tend to consider other therapeutic modalities than prostate removal in very obese patients. With the surge in robotic surgery in the last two decades, more obese patients have undergone robot-assisted radical prostatectomy (RARP). Objective: This current, monocentric, retrospective serial study investigates primarily the impact of obesity on readmissions and secondarily the major complications of RARP. Methods: Five hundred patients from one referral center who underwent RARP between April 2019 and August 2022 were included in this retrospective study. To investigate the impact of patient BMI on postoperative outcomes, we divided our cohort into two groups with a cut-off of 30 kg/m^2^ (according to the WHO definition). Demographic and perioperative data were analyzed. Postoperative complications and readmission rates were compared between standard, normal patients (NOBMI—BMI under 30; n = 336, 67.2%) and overweight patients (OBMI—BMI equal to/more than 30; n = 164, 32.8%). Results: OBMI patients had bigger prostates on TRUS, more comorbidities and worse baseline erectile function scores. They also received fewer nerve-sparing procedures than their counterparts (*p* = 0.005). Analysis showed no statistically significant differences in readmission rates or in minor or major complications (*p* = 0.336, 0.464 and 0.316, respectively). In a univariate analysis, BMI could predict positive surgical margins (*p* = 0.021). Conclusion: Performing RARP in obese patients seems to be safe and feasible, without major adverse events or elevated readmission rates. Obese patients should be informed preoperatively about the elevated risk of higher PSMs and technically more difficult nerve-sparing procedures.

## 1. Introduction

Due to more difficult intraoperative courses, elevated rates of case abortion and unfavored postoperative outcomes [1,2], urologists tend to locally treat obese patients with radiation rather than surgical therapy. An association between overweight status, among other baseline parameters, with longer OR time and greater blood loss is well acknowledged [3]. With the surge in robotic surgery, more obese patients are being offered robot-assisted radical prostatectomy (RARP). To overcome the obesity-related poorer perioperative outcomes and to improve cancer-related survival, the effect of weight loss in overweight patients prior to RARP was examined [4]. Jointly, low-calorie diets and exercise programs resulted in reductions in regional fat mass with improvements in blood pressure, which may benefit surgical outcomes [4]. Higher body mass index (BMI) combined with an aggressive tumor also increases the risk of symptomatic lymphoceles [5]. Obesity in patients undergoing RARP is additionally associated with increased operating times, fewer nerve-sparing procedures and higher rates of positive surgical margins (PSMs) [6,7,8]. However, the effect of BMI in a monocentric, single-surgeon cohort study of 500 patients, one-third of whom were obese and 40% of whom had locally advanced tumors, on readmissions and major postoperative complications specifically has not been thoroughly investigated. Our current study aimed to investigate the impact of obesity from a clinical point of view using a large in-hospital database.

## 2. Methods

### 2.1. Surgical Procedure and Setting

All procedures (n = 500) were carried out transperitoneally with the Da Vinci X^®^ Surgical System (Intuitive Surgical, Sunnyvale, CA, USA). Extended pelvic lymphadenectomy was performed in all patients, and no intra-abdominal drain was inserted. Prior to skin incision, intravenous single-shot antibiotics were administered. The vesicourethral anastomosis (VUA) was performed in a one-layer fashion with a continuous, circumferential, double-armed barbed suture. In most cases, the anastomosis included a one-layer Rocco stitch. After completion, patients underwent an intraoperative anastomosis water-tightness test with 200–300 mL of sterile water. All patients received a transurethral (TUC) and a suprapubic catheter (SPC). The transurethral catheter was removed on the first postoperative day (POD1). On POD3, patients were allowed to urinate naturally. When they did not report any micturition disorders, the suprapubic catheter was removed after one day. In case of primary extravasation on cystography, patients were discharged with catheters, and catheters were removed during outpatient visits a couple of days later. 

### 2.2. Participants and Methods

Five hundred consecutive patients from a prospectively collected database who underwent RARP between April 2019 and August 2022 performed by a specialized surgeon for locally confined (pT2; n = 295, 59.4%) and locally advanced prostate cancer (pT3-4; n = 203, 40.6%) were included in this analysis. 

According to their body mass index (BMI), the patients were divided into groups based on a cut-off value of 30 kg/m^2^: a normal group (NOBMI) with BMIs under 30 kg/m^2^ and an overweight group (OBMI) with BMIs equal to or more than 30 kg/m^2^. Demographic, intraoperative and postoperative data were analyzed and compared with propensity-score matching: NOBMI vs. OBMI (2:1). The variables included were age, International Prostate Symptom Score (IPSS), International Index of Sexual Function (IIEF), initial PSA, pre- and postoperative Gleason scores, prostate volume in transrectal ultrasound (TRUS), American Association of Anesthesiology (ASA) morbidity score, pre- and postoperative hemoglobin (Hgb), previous medical and surgical treatment of the prostate, and D’Amico risk classification. Postoperative complications were graded according to the Clavien–Dindo classification system [9]. The primary endpoint was the difference in readmissions, and the secondary endpoint was the difference in the incidence of complications between the two groups. A univariate linear regression analysis to examine whether BMI could predict readmissions or other postoperative outcomes was carried out to determine which parameters are most affected most by obesity. 

Statistical analysis was performed using SPSS v27. Categorical variables were summarized as frequencies (percentages) and continuous variables as means ± standard deviations, interquartile ranges (IQRs) and median values. The Kolmogorov–Smirnov one-sample test was used to verify normal distributions. Matched-pair analysis using an independent *t*-test for parametric numeric variables and the Mann–Whitney U-Test for nonparametric variables was performed. Pearson’s chi-squared test was also used to compare relative frequencies. Univariable logistic regression and linear regression models were used in the analysis. 

### 2.3. Ethics Statement

The study was conducted in accordance with the ethical standards of the Declaration of Helsinki and approved by the ethics committee of the Medical Association of Westfalen-Lippe and Wilhelm’s University of Münster (2022-585-f-S).

## 3. Results

Baseline Parameters: Baseline clinical and preoperative characteristics were comparable between groups. Higher American Association of Anesthesia (ASA) scores, specifically ASA 3 (10.1 vs. 29.3%), were observed more in obese patients (*p* < 0.001). Additionally, OBMI patients had bigger prostates on TRUS (*p* = 0.002). Obese patients had worse baseline erectile function IIEF scores (*p* = 0.001). Neurovascular bundles were spared less frequently in OBMI men than in their NOBMI counterparts (*p* = 0.005). Details are given in Table 1. 

Intraoperative data: Analysis showed no statistical difference in console operating time, with a median of 140 min (*p* = 0.760). Obese patients had bigger prostates in the final pathological report (median: 62 vs. 52 g; *p* = 0.009). Oncological results were similar between groups regarding tumor stage, Gleason score distribution and positive surgical margins, as well as positive lymph nodes. Notably, the number of total lymph nodes removed was higher in obese patients (median: 19 vs. 18; *p* = 0.008). Catheter days and length of hospital stay were similar between the groups (*p* = 0.092 and 0.438, respectively). A patient with severe LUTS, a very big prostate and low PSA was referred to our department for robot-assisted simple prostatectomy. In the final pathology, a T1b Gleason 3 + 3 = 6 prostate carcinoma was reported. After discussing all treatment possibilities with the patient, he opted for radical prostatectomy, which we performed uneventfully. In the second pathology, no prostate cancer was found, and the tumor was staged by our pathologists as T0. Details are given in Table 2. 

Complications: Both minor and major complications occurred equally in both groups (*p* = 0.464 and 0.316, respectively). Clavien–Dindo I includes thromboembolic incidents, elevated laboratory tests and acute urinary retention, among other minor complications requiring beside medication no medical interventions. Secondary vesicourethral anastomosis leakage (VUAL) at discharge was rare (n = 11; 2.2%), with similar incidences between the groups. These patients had catheters removed at an outpatient visit with unproblematic micturition, without elevated residual urine. Urinary tract infections were treated with oral or parenteral antibiotics depending on the clinical manifestation (n = 11, 2.2%). Major complications can be divided into two main categories. The first of these is Grad IIIa, which represents interventions carried out with local anesthesia, mostly therapy of a symptomatic lymphocele (n = 10; 2%). In these cases, a drain was inserted percutaneously and left in place for several days. Trends suggested a higher incidence in the OBMI vs. the NOBMI group (3% vs. 1.4%). One patient with a long history of coronary heart disease and an uneventful intra- and postoperative course presented one week after discharge with an NSTEMI and received a PTCA with coronary stents, with an uneventful further clinical course. Patients who experienced the second category of complications, Grad IIIb, included those who had to be operated on under general anesthesia for bleeding (n = 1), incisional hernia (n = 2) or bowel obstruction (n = 1). Three men experienced upper urinary tract obstruction (UTTO; n = 3, 0.6%), in which a DJ catheter was inserted retrogradely. One patient (BMI: 35) with a T4 tumor showed signs of rhabdomyolysis and was therefore admitted to the intensive care unit for hemodialysis. His symptoms were temporary, and he recovered fully with no permanent lesions. Details are given in Table 3.

Readmission rate: After discharge, n = 28 (5.6%) of patients were readmitted within 90 days after RARP. No differences were observed between groups: n = 21 (6.25%) patients in the NOBMI group were readmitted vs. n = 7 (4.2%) in the OBMI group (*p* = 0.336). 

A univariate analysis for BMI could not solely predict those patients at risk of readmission (*p* = 0.221) or who may experience major complications (*p* = 0.200), nor the length of hospital stay, catheter days or incidence of lymphoceles (*p* = 0.317, 0.225 and 0.637, respectively). The only correlation to be found was with positive surgical margins, these being significantly higher in the OBMI group (*p* = 0.021). Details are given in Table 4.

## 4. Discussion

The main result of our study is that, with adequate surgical experience, operating on obese patients seems to be safe and feasible since it does not put them at elevated risk of suffering major complications or more readmissions after RARP. Furthermore, obese patients received fewer nerve-sparing procedures compared to their counterparts (61% vs. 73.5%; *p* = 0.005). They did not suffer greater blood loss. They also did not undergo longer operations. Regarding the oncological aspects of our results, positive surgical margins were equal between the groups. 

Generally, prostatectomy in obese patients is linked with increased perioperative morbidity [10]. With the surge in robot-assisted radical prostatectomy (RARP), more obese patients are being considered for robot-assisted radical prostatectomy (RARP). Remarkably, at the beginning of the robotic era, operating on obese patients was associated with poorer perioperative and long-term outcomes [11,12]. In total, n = 28/500 (5.6%) patients were readmitted within 90 days after RARP. This accords with other large series reporting 3.3% readmissions within 30 days after discharge [13]. It is also similar to rates among patients without obesity stratification [14]. Our moderately elevated rate is explainable partially because we reported 90-day readmissions. Secondly, the proportion of obese men in our series was almost one-third, which is significantly higher than in other series, for which the figure was 8.6% [15]. Interestingly, fewer obese patients were readmitted after discharge (4.2%) than normal and overweight patients (6.25%), with no statistical difference exposed (*p* = 0.336). Despite the fact that one-third of our patients were obese and that 40% of them had locally advanced tumors, our results are comparable to those of other cohort studies [13]. The incidences of both minor and major complications were equal between the groups (*p* = 0.464 and 0.316, respectively). This is in accordance with the findings of other authors (Xu et al., 2015) [1,8,16]. These comparable outcomes for normal-weight and obese patients might be explained by many factors. Firstly, there is the robotic approach that has become well established in prostate surgery over the last decade, combined with the high volume of well-trained surgeons with structured learning curves even earlier in their careers. Additionally, there is the standardization of procedures and the constant increase in institutional experience. It ought to be mentioned that this was a large clinical study including 500 patients, reflecting real-world data, with a third of the cohort being obese (BMI > 30 kg/m^2^), without any exclusion criteria applied, and which reported the readmissions for any complication within 90 days after discharge. 

As expected, nerve-sparing procedures were carried out less frequently in obese patients (61% vs. 73.5%; *p* = 0.005). Abdul-Muhsin et al. reported similar findings for their cohort of obese (BMI > 40 kg/m^2^) patients (*p* = 0.097) [8]. Preoperatively, 30.8% of men were D’Amico high-risk cases, which was confirmed postoperatively, as 40% of patients had extra-prostatic tumor extension. Additionally, a considerable proportion of men in our study presented with definite erectile dysfunction. This may explain the limited number of nerve-sparing procedures performed in our cohort. Nonetheless, we managed to spare the nerves in n = 374/500 (69.4%) of men, and in n = 19/500 (3.8%) a partial nerve-sparing procedure was performed. The obese patients in our cohort, as elsewhere, had a worse baseline erectile function score (17 vs. 12; *p* = 0.001), which does not necessitate the performance of a nerve-sparing procedure. They also had more concomitant comorbidities and therefore usually worse general-status ASA scores (*p* < 0.001) [1]. To measure the impact of obesity on intraoperative blood loss, urologists considered the estimated blood loss and transfusion rates. To be more specific, we compared the difference in hemoglobin before surgery and before discharge. Similar to other authors [6], we did not find a difference between the groups (*p* = 0.082). The same goes for transfusion rates (*p* = 0.162). Others found obese patients to lose more blood intraoperatively due to more difficult intraoperative courses [16,17]. 

Considering EAU guidelines [18], a pelvic lymph adenectomy has to be performed when the likelihood of lymph node metastases exceeds 5%. This can be anticipated through various nomograms, such as Briganti’s [19] or Partin’s [20]. Performance of a pelvic lymph adenectomy is associated with longer procedures; elevated intraoperative complications, such as vessel injuries; and postoperative complications, such as symptomatic lymphoceles [21,22,23]. Nonetheless, we completed pelvic lymph adenectomies in all our patients regardless of their clinical stage or pathological grade to avoid the risk of under-staging or under-grading because of the high rate of tumor misclassification by referring urologists. Moreover, more than 20% of men in our current study proved to have positive lymph nodes. This figure is significantly higher than those given in previous reports [19]. However, the incidence of symptomatic lymphoceles in our cohort remained at 2% (n = 10/500), despite relatively extended lymph adenectomies (a median of 18 lymph nodes per patient). These complications were of a mild nature and were exclusively treated by insertion of a drain with local anesthesia administered in most cases. Furthermore, in a tertiary institution such as the one in which the study was performed, the diagnostic work-up and biopsies are carried out externally. This may have influenced the homogeneity of the pathological reports for our patients and may indicate uncertainty regarding their staging and grading.

Whereas the majority of other studies reported longer operating times for obese patients [1,17], the median console time in our cohort was 140 min for both groups (*p* = 0.760). This might also explain the low rate of complications, such as thromboses, embolies or complications related to patient positioning, in our cohort. Diverse reports describe the influence of BMI on positive surgical margins (PSM). We noticed a trend for more PSMs in our obese patients (10.4 vs. 5.7%). Whereas others found obese patients to endure more positive margins [2,8], our analysis showed no statistically significant difference (*p* = 0.056), similar to the findings of Albisini et al. [6,7]. The number of lymph nodes dissected in obese patients was also higher (19 vs. 18; *p* = 0.008). Despite a trend of more positive lymph nodes being detected in obese patients (20.1 vs. 16.1%), statistical analysis showed no difference (*p* = 0.262). 

Obese patients possessed larger prostates compared to NOBMI patients (47 vs. 41 mL on TRUS). This was also confirmed for prostate weight in final pathological reports (62 g for OBMI vs. 52 g for NOBMI). As we showed in a previous study [21], prostate volume can prolong catheter days and increase the incidence of minor complications, such as acute urinary retention. However, prostate volume has less influence on major complications, readmissions and oncological results. In our current study, although the difference in prostate volume between groups was statistically significant, it had no clinical consequences.

While Goßler et al. reported in their multivariate analysis that obesity did not correlate with PSMs, they stated also that only preoperative prostate-specific antigen (PSA) levels and pathological tumor stage had a significant effect on PSMs [2]. We found in a univariate linear regression analysis including many variables that BMI was a positive predictor only for positive surgical margins (*p* = 0.021). 

The strength of our investigation is the large number of patients included and the detailed analysis of their perioperative parameters and outcomes. However, certain limitations must be taken into account. The main limitation of our study is its retrospective nature. Second, we could not include long-term outcome measures due to the lack of follow-up data, which is partly explained by the national health care system, in which follow-ups are not conducted by tertiary referral centers. The relevance of our study and therefore its evidence base is relatively narrow, since it was solely based on a high-volume urological department and an expert surgeon. Moreover, among patients undergoing RARP, differences in outcomes are associated with patient complexity. Our cohort included a high proportion of locally advanced tumors (40.6%). Still, obese patients underwent the same safe, uneventful intra- and postoperative course of RARP in 96% of cases. Only 5.6% of patients in total were readmitted, more frequently at the beginning of our study, which implicates an institutional learning curve. However, more than 70% received a nerve-sparing procedure without compromise to the oncological aspect of the procedure, with negative surgical margins in more than 93% of men. 

## 5. Conclusions

A total of 96% of men undergoing RARP regardless of their habitus status would be uneventfully discharged. In experienced hands, operating on obese patients seems to be safe and feasible without elevated risk for major adverse events or readmission rates. These patients must be informed preoperatively about the relatively lower possibility of performing nerve-sparing procedures due to more difficult operative courses, and particular care must be taken to avoid positive surgical margins. In addition, weight loss regimens have to be recommended preoperatively to maintain good postoperative results. 

## Figures and Tables

**Table 1:** Analysis of demographic, baseline clinical and preoperative characteristics.

	Total (500)	NOBMI (n = 336, 67.2%)	OBMI (164, 32.8%)	*p*-Value
Age (year)				
Mean ± SD IQR Median	66.8 ± 7.1 62–72 68	66.75 ± 6.6 61.25–72 68	66.95 ± 8 62–72 68	0.769
ASA score				
1 2 3 Missing	96 (19.2) 314 (62.8) 82 (16.4) 8 (1.6)	81 (24.1) 219 (65.2) 34 (10.1) 2 (0.6)	15 (9.1) 95 (57.9) 48 (29.3) 6 (3.6)	>0.001
Preoperative HGB (g/dL)				
Mean ± SD IQR Median	14.7 ± 1.3 14.1–15.5 14.8	14.7 ± 1.26 (14.1–15.5) 14.8	14.65 ±1.38 (13.9–15.5) 14.8	0.491
IPSS				
Mean ± SD IQR Median	11.4 ± 8.3 5–16 8.3	11.43 ± 8.2 (5–16) 10	11.4 ± 8.4 (4–17.5) 10	0.437
IIEF				
Mean ± SD IQR Median	15.2 ± 8.7 6–23 17	16.3 ± 8.5 8–24 17	13.1 ± 8.5 8–24 12	0.001
Initial PSA (ng/mL)				
Mean ± SD IQR Median	14.8 ± 24.5 5.5–13.6 8	14.4 ± 23.8 5.4–12.6 7.7	15.6 ± 26 5.6–14.9 9	0.607
Prostate Volume (mL)				
Mean ± SD IQR Median	49 ± 28 31–60 43	46 ± 23.1 31–56 41	55 ± 37 31–56 47	0.002
Pretreatment				
Medical Surgical	55 (11) 34 (6.8)	29 (8.6) 22 (6.5)	26 (15.8) 12 (7.3)	0.050 0.856
D’Amico Risk Classification				
Low Risk Intermediate Risk High Risk	117 (23.4) 229 (45.8) 154 (30.8)	90 (26.8) 145 (43.2) 101 (30.1)	27 (16.5) 84 (51.2) 53 (32.3)	0.083
Preoperative Gleason Score				
5 6 3 + 4 4 + 3 8 9 10 Unclassified *	1 (0.2) 140 (28) 176 (35.2) 59 (11.8) 82 (16.4) 36 (7.2) 5 (1.0) 1 (0.2)	1 (0.3) 105 (31.3) 112 (33.3) 38 (11.3) 54 (16.1) 22 (6.5) 3 (0.9) 1 (0.3)	35 (21.3) 64 (39) 21 (12.8) 28 (17.1) 14 (8.5) 2 (1.2)	0.327
Nerve Sparing				
Yes Partial No	374 (69.4) 19 (3.8) 134 (26.8)	247 (73.5) 13 (3.9) 76 (22.6)	100 (61) 6 (3.7) 58 (35.4)	0.005

Categorical data are presented as numbers (%). * Patients received androgen deprivation therapy preoperatively. SD: standard deviation, IQR: interquartile range, ADT: androgen deprivation therapy, ASA: American Association of Anesthesiology comorbidity score, HBG: hemoglobin, BMI: body mass index, IPSS: International Prostate Symptom Score, IIEF: International Index of Erectile Function, NOBMI: group of patients with BMI under 30 kg/m^2^, OBMI: obese patients with BMI equal or more than 30 kg/m^2^, PSA: prostate-specific antigen, TUR-P: transurethral resection of the prostate.

**Table 2:** Intra- and postoperative data and pathological findings.

	Total (500)	NOBMI (n = 336, 67.2%)	OBMI (164, 32.8%)	*p*-Value
Console Time (Minute) Mean ± SD IQR Median	151 ± 45 120–180 140	150 ± 47 120–178 140	152 ± 41 120–178 140	0.760
Prostate Weight (g) Mean ± SD IQR Median	61 ± 25.6 64–72 55	58 ± 22.9 43.7–69 52	68 ± 29 50–80.5 62	0.009
Pathological Stage 0 pT1 pT2 pT3 pT4	1 (0.2) 1 (0.2) 295 (59) 183 (36.6) 20 (4.0)	212 (63) 112 (33.3) 12 (3.6)	1 (0.6) 1 (0.6) 83 (50.6) 71 (43.2) 8 (4.8)	0.088
Postoperative Gleason Score 6 3 + 4 4 + 3 8 9 10 Unclassified *	28 (5.6) 282 (56.4) 89 (17.8) 26 (5.2) 29 (5.8) 1 (0.2) 45 (9.0)	22 (6.5) 195 (58) 59 (17.6) 18 (5.4) 16 (4.8) 1 (0.3) 25	6 (3.7) 87 (53) 30 (18.3) 8 (4.9) 13 (7.9) 20	0.284
Positive Surgical Margins (Total) <3 mm >3 mm	36 (7.2) 17 (3.4) 19 (3.8)	19 (5.7) 10 (2.9) 9 (2.6)	17 (10.4) 7 (4.2) 10 (6.1)	0.056
Number of Lymph Nodes Mean ± SD IQR Median	19.6 ± 7.4 (15–24) 18	19 ± 7 14–23 18	20.9 ± 8 16–26.7 19	0.008
Positive Lymph Nodes	87 (17.4)	54 (16.1)	33 (20.1)	0.262
HGB Difference (g/dL) Mean ± SD IQR Median	2.5 ± 4.8 1.9–3.5 2.6	2.7 ± 1.3 2.0–3.6 2.7	1.9 ± 8.1 1.7–3.4 2.5	0.082
Transfusion	7 (1.2)	5 (1.4)	2 (1.2)	0.162
Hospitalization (days) Mean ± SD IQR Median	5.6 ± 1.5 5–6 5	5.5 ± 1.2 5–6 5	5.7 ± 2.1 5–6 5	0.438
Catheter Days Mean ± SD IQR Median	6.9 ± 4.7 4–10 5	6.89 ± 4.7 4–10 5	7.03 ± 4.6 4–10 5	0.092

Categorical data are presented as numbers (%). * Patients received androgen deprivation therapy preoperatively. SD: standard deviation, IQR: interquartile range, HGB: hemoglobin, NOBMI: group of patients with BMI under 30 kg/m^2^, OBMI: obese patients with BMI equal or more than 30 kg/m^2^.

**Table 3:** Ninety-day complication and readmission rates for the two groups.

			Total (n = 500)	NOBMI (n = 336, 67.2%)	OBMI (164, 32.8%)	*p*-Value
Minor	CDI 51 (10.2)	Thrombus/Embolism	4 (0.8)	2 (0.6)	2 (1.2)	0.464
		Elevated Labor Parameter	6 (1.2)	1 (0.3)	5 (3.0)	
		AUR	18 (3.6)	10 (2.9)	8 (5.7)	
		Diverse	13 (2.6)	7 (2.0)	6 (3.6)	
	CD II 23 (4.6)	Secondary VUAL *	11 (2.2)	8 (2.3)	3 (1.8)	
		UTIs	11 (2.2)	8 (2.3)	3 (1.8)	
		Hematoma Requiring Transfusion	1 (0.2)	1 (0.3)	0	
Major	CD III a 12 (2.4)	Myocardial Infarction	1 (0.2)	1 (0.3)	0	0.316
		Hiatus Hernia	1 (0.2)	1 (0.3)	0	
		Symptomatic Lymphocele	10 (2.0)	5 (1.4)	5 (3.0)	
	CD III b 8 (1.6)	Revision	5 (1.0)	3 (0.9)	2 (1.2)	
		UUTO	3 (0.6)	2 (0.6)	1 (0.6)	
	CD IV 1 (0.2)	Rhabdomyolysis	1 (0.2)	1 (0.3)	0	
		Readmissions	28 (5.6)	21 (6.25)	7 (4.2)	0.336

* Readmissions do not correlate linearly with complications, since some major complications happened predischarge. CD: Clavien–Dindo, UTTO: upper urinary tract obstruction, AUR: acute urinary retention, VUAL: vesicourethral anastomosis leakage.

**Table 4:** Univariate linear regression analysis of BMI to predict for readmissions and other postoperative outcomes.

	Readmission	Major Complications	Catheter Days	Hospital Stay	Lymphoceles	Positive Surgical Margins	Transfusion
BMI	0.221	0.200	0.225	0.317	0.673	0.021	0.234

## Data Availability

Data supporting results is available when requested.

## Abbreviations

ADT	Androgen deprivation therapy
ASA	American Association of Anesthesiology comorbidity score
AUR	Acute urinary retention
BMI	Body mass index
CD	Clavien–Dindo classification of postoperative complications
Hgb	Hemoglobin
IIEF	International Index of Erectile Function
IPSS	International Prostate Symptom Score
NHT	Neoadjuvant hormonal therapy
NSTEMI	Non-ST-segment elevation myocardial infarction
NOBMI	BMI under 30
OBMI	BMI equal to/more than 30
POD	Postoperative day
PSA	Prostate-specific antigen
PSM	Positive surgical margin
TUR-P	Transurethral resection of the prostate
RARP	Robot-assisted radical prostatectomy
SPC	Suprapubic catheter
TUC	Transurethral catheter
LOS	Length of hospital stay
UTI	Urinary tract infection
VTE	Venous thromboembolism
UUTO	Upper urinary tract obstruction
VUA	Vesicourethral anastomosis
VUAL	Vesicourethral anastomosis leakage
SVUA	Secondary vesicourethral anastomosis leakage
